# Plant structural biology: Emerging technologies and future biological insights

**DOI:** 10.1111/tpj.70786

**Published:** 2026-03-11

**Authors:** Jonas M. Böhm, Veronica G. Maurino

**Affiliations:** ^1^ Molecular Plant Physiology, Institute for Cellular and Molecular Botany (IZMB) University of Bonn Kirschallee 1 53115 Bonn Germany

**Keywords:** AI‐based structure prediction, cryo‐electron microscopy, emerging technologies, plant structural biology, structural modelling

## Abstract

Plant structural biology is entering a new era. Advances in cryo‐electron microscopy, tomography, and AI‐based prediction are making it possible to study plant macromolecular machines at near‐atomic resolution, including complexes that long resisted analysis by traditional approaches. Yet, despite these developments, plant proteins remain underrepresented in structural databases, reflecting persistent challenges in sample preparation, *in situ* imaging, and capturing dynamics. At the same time, plants present unique opportunities for structural biology, from the photosynthetic apparatus and cellulose synthase rosettes to receptor‐like kinases, resistosomes, and plastid protein import machinery. Understanding these systems requires not only technical innovation but also conceptual shifts toward structural landscapes that capture molecular heterogeneity across time, space, and environmental conditions. Here, we outline the main frontiers for the field: improving sample preparation pipelines, advancing *in situ* and time‐resolved methods, integrating structural biology with omics, and harnessing computational modeling. We highlight biological questions where structural insights are most urgently needed, including photosynthesis, hormone signaling, cell wall synthesis, organelle biology, and immunity. We argue that investment in infrastructure, training, and collaborative networks is essential if plant structural biology is to realize its potential. By revealing the molecular logic of the green world, the field can contribute solutions to urgent challenges in agriculture, sustainability, and climate resilience.

## Significance Statement

Structural biology is beginning to reveal how plant molecular machines work, yet plant proteins remain underrepresented compared with biomedical targets. With advances in cryo‐EM, tomography, and AI‐based modeling, it is now possible to study complex, dynamic, and plant‐specific assemblies in unprecedented detail, paving the way from static protein snapshots to structural landscapes with transformative implications for crop improvement, stress resilience, and sustainability.

## PLANT STRUCTURAL BIOLOGY AT A TURNING POINT

**Figure 1:**
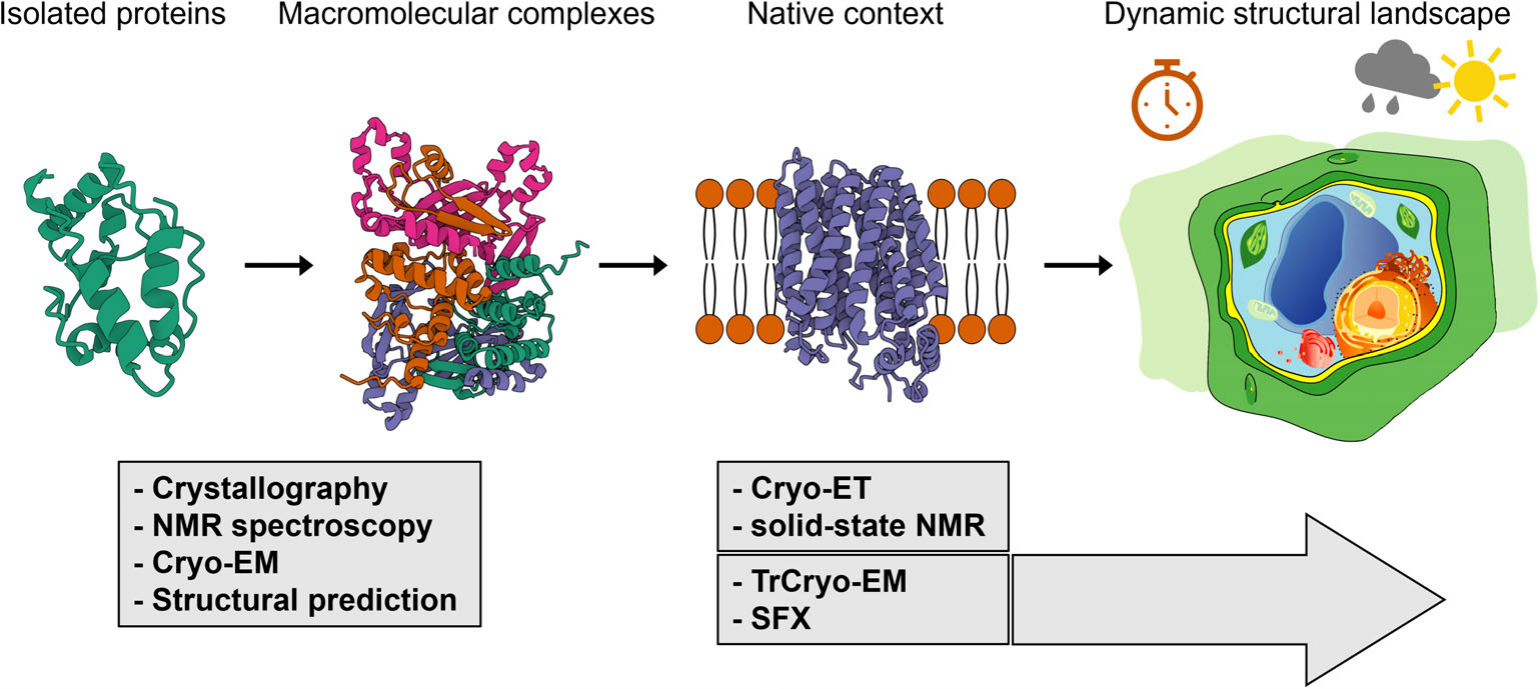
Structural landscapes framework. Structural biology is evolving from static snapshots of individual proteins to multidimensional “structural landscapes” that capture dynamics, spatial context, and environmental responsiveness across scales. Examples of methods that provide high‐resolution structures of proteins and multi‐subunit complexes include X‐ray crystallography, nuclear magnetic resonance (NMR) spectroscopy, cryo‐electron microscopy (cryo‐EM), and AI‐based structure prediction. Native and near‐native states can be probed by approaches such as cryo‐electron tomography (cryo‐ET) and solid‐state NMR spectroscopy. Techniques like time‐resolved cryo‐EM (TrCryo‐EM) and serial femtosecond crystallography (SFX) further add a temporal dimension, enabling the visualization of biological reactions and systems *in situ*. Together, these and other emerging approaches pave the way toward dynamic structural landscapes that integrate molecules, cells, and their environments.

Structural biology has long been central to biology, providing the molecular blueprints that underpin mechanistic understanding. For decades, X‐ray crystallography and nuclear magnetic resonance (NMR) spectroscopy dominated the field, yielding atomic structures of soluble proteins and smaller macromolecules (Figure 1). Yet, despite their power, these approaches had clear limitations when applied to the plant kingdom. Many plant proteins are large, membrane‐bound, or form dynamic assemblies that resist crystallization, while others carry complex post‐translational modifications that complicate heterologous expression and purification. As a result, structural insights into plant proteins have historically lagged behind those from microbial or animal systems.

Nonetheless, pioneering studies in plant structural biology have had a profound impact on the field as a whole. One of the earliest and most emblematic successes was the crystallographic analysis of ribulose‐1,5‐bisphosphate carboxylase/oxygenase (Rubisco), which revealed the architecture of the world's most abundant enzyme and provided the basis for decades of research on photosynthetic carbon fixation (Knight et al., 1990; Taylor & Andersson, 1997). Similarly, the elucidation of photosystem I and II complexes by X‐ray crystallography in the 1990s and early 2000s represented watershed moments, providing the first high‐resolution glimpses into the molecular machines that drive light harvesting and water splitting (Ben‐Shem et al., 2003; Ferreira et al., 2004; Jordan et al., 2001). These structures not only deepened our understanding of photosynthesis but also established plants and cyanobacteria as legitimate systems for cutting‐edge structural work. Crystallography and later advances in NMR spectroscopy allowed structural studies of plant hormone receptors and signaling components, including the perception mechanism of auxin by TIR1/AFB proteins and the recognition of jasmonate by the COI1 receptor (Han et al., 2014; Sheard et al., 2010; Tan et al., 2007). Each of these milestones demonstrated that plant‐specific processes could be understood at atomic detail, but they also highlighted the exceptional effort required to achieve such breakthroughs.

Despite these achievements, plant proteins remain underrepresented in the Protein Data Bank (PDB) (Maurino, 2025). A large fraction of deposited plant structures corresponds to conserved metabolic enzymes or proteins expressed in microbial systems, while truly plant‐specific assemblies such as cellulose synthase complexes, receptor‐like kinases (RLKs), or plastid protein import machinery are still poorly represented. The imbalance reflects both technical and sociological factors. Technically, many plant proteins are difficult to overexpress, prone to heterogeneity, or embedded in membranes and cell walls. Sociologically, structural biology has historically been driven by biomedical imperatives, which naturally prioritized human proteins, pathogens, and drug targets. By contrast, the structural biology of plant proteins has often been viewed as a niche pursuit, despite its broad implications for agriculture, ecology, and sustainability.

The so‐called “resolution revolution” in cryo‐electron microscopy (cryo‐EM) has begun to change this situation (Bai et al., 2015; Callaway, 2020; Kuhlbrandt, 2014). In less than a decade, cryo‐EM has transitioned from a niche method to a mainstream technique capable of routinely delivering near‐atomic structures of large macromolecular complexes (Figure 1). Its ability to visualize proteins without crystallization has been particularly transformative for membrane proteins, supramolecular assemblies, and complexes in multiple functional states. Alongside cryo‐EM, complementary developments in cryo‐electron tomography (cryo‐ET) (Lamm et al., 2022; Liedtke et al., 2022; Nogales & Mahamid, 2024; Sanchez Carrillo et al., 2023) and X‐ray free electron lasers (XFELs) (Liu & Lee, 2019; Martin‐Garcia et al., 2016) are expanding the range of structures and dynamics that can be captured (Figure 1). Computational advances, especially the advent of AI‐driven structure prediction tools such as AlphaFold (Abramson et al., 2024; Chen, Zia, et al., 2024; Jumper & Hassabis, 2022) and RoseTTAFold (Baek et al., 2021; Chen, Li, et al., 2024), have further broadened the scope of structural biology, even providing models for entire proteomes (Seong & Krasileva, 2023; Varadi et al., 2023; Wang et al., 2025; Woodhouse et al., 2023) (Figure 1).

At the same time, plants themselves offer unique opportunities for structural biology. Their physiology and development depend on molecular machines with no equivalents in animals or microbes, including the photosynthetic apparatus, cellulose synthase rosettes, and plant‐specific immune receptors. Many of these assemblies are dynamic, membrane‐embedded, and tightly regulated by environmental conditions, making them both challenging and exciting structural targets. Moreover, plants exhibit remarkable cellular complexity, with plastids, vacuoles, plasmodesmata, and cell walls providing additional layers of organization that extend well beyond the protein scale. Understanding plant biology at the structural level therefore demands methods that bridge from molecules to organelles, cells, and tissues.

The present moment offers a rare convergence of enabling technologies and pressing global needs. On the one hand, technical barriers to high‐resolution structure determination are falling, as cryo‐EM, tomography, and AI modeling become more widely accessible. On the other hand, the climate crisis, biodiversity loss, and rising demands on agriculture highlight the urgency of understanding plant biology at a mechanistic level. Structural biology can provide a foundation for strategies to improve photosynthetic efficiency, engineer stress tolerance, and optimize cell wall composition for bioenergy and biomaterials. In this sense, plant structural biology is not simply a branch of basic science but a field with the potential to contribute solutions to some of the most urgent challenges of the 21st century. This perspective article highlights the main questions that define the future of plant structural biology, focusing on both technological frontiers and biological insights.

## TECHNOLOGICAL FRONTIERS

The progress of plant structural biology is closely tied to advances in methodology. While cryo‐EM and AI‐based modeling have democratized access to high‐resolution structures, plants present specific technical challenges that require further innovation. Overcoming these hurdles will be essential for the field to advance from isolated case studies to a systematic understanding of plant macromolecular assemblies.

### Sample preparation: The persistent bottleneck

Perhaps the greatest practical challenge in plant structural biology remains sample preparation. Many of the most important plant proteins are embedded in membranes, expressed at low abundance, or extensively modified. Heterologous expression often fails to reproduce correct folding and post‐translational modifications, while purification is frequently hindered by instability or heterogeneity. Advances in transient expression (Opdensteinen et al., 2022), lipid‐mimicking systems such as nanodiscs (Henrich et al., 2017; Lalli et al., 2017; Warschawski et al., 2011), and direct extraction of native complexes offer promising directions, but plant‐tailored pipelines remain to be established.

### *In situ* approaches: Seeing molecular machines in context

Purified structures remain indispensable, but many plant processes depend on the organization of complexes within their native environment (Figure 1). Recent advances in cellular solid‐state NMR spectroscopy have resolved membrane protein structures at atomic resolution *in situ* (Xie et al., 2023), opening the way to studies of more complex systems, including plant membranes. Complementary to this, cryo‐ET offers the ability to visualize assemblies directly in cells and organelles (Nogales & Mahamid, 2024). Studies of thylakoid membranes and plasmodesmata hint at the potential of these methods to reveal organization that is lost *in vitro* (Dickmanns et al., 2025; Engel et al., 2015; Wietrzynski et al., 2025). However, the large size and rigidity of plant cells pose challenges for sample preparation, requiring innovations such as cryo‐focused ion beam milling (Sanchez Carrillo et al., 2023). Developing workflows that make *in situ* structural biology routine for plants will be essential to unlock this next frontier.

### Capturing dynamics: Beyond static snapshots

Plant processes, such as photosystem repair, hormone perception, and immune activation are dynamic by nature. Static structures capture only one state, leaving unanswered how transitions occur. Time‐resolved methods, such as time‐resolved cryo‐EM (TrCryo‐EM; Lorenz, 2024) or serial femtosecond crystallography (SFX; Martin‐Garcia et al., 2016), are beginning to reveal transient intermediates in photosynthetic reactions and chromophore isomerization (Claesson et al., 2020; Kupitz et al., 2014; Li et al., 2021), but their application to plant signaling or stress responses is still rare. Establishing pipelines for time‐resolved studies will allow researchers to move toward molecular “movies” of plant biology, capturing not only what complexes look like but how they work (Figure 1).

### Integration with omics: Linking molecules to systems

Plant biology is increasingly shaped by large‐scale omics approaches, from single‐cell transcriptomics to proteomics and metabolomics. Structural biology has much to gain by integration with these resources. Proteomics can guide target selection and reveal native complexes, while cross‐linking or co‐fractionation data provide interaction maps that complement structural studies. Spatial transcriptomics and proteomics can highlight tissue‐ or organ‐specific expression, suggesting where and when to seek particular structures *in situ* (Breckels et al., 2024; Olanrewaju et al., 2023). In the long run, the challenge will be to weave molecular structures into multiscale frameworks that connect genotype to phenotype.

### Computational tools: Promises and gaps

AI‐driven structure prediction has transformed structural biology, producing accurate models for thousands of plant proteins and protein complexes (Chaaban et al., 2024; O'Reilly et al., 2023) (Figure 1). These predictions are already accelerating hypothesis generation and providing templates for interpreting low‐resolution data. Yet, limitations remain, particularly for large multi‐subunit complexes and intrinsically disordered regions. Moreover, predicted models lack context: lipids, cofactors, and modifications that are central to plant protein function are generally missing. Bridging this gap between *in silico* predictions and biological reality will depend on iterative cycles of prediction, experiment, and refinement.

## BIOLOGICAL CHALLENGES

Technological advances are opening the way for structural studies of plants, but the most compelling questions are ultimately biological. Plants harbor molecular machines that are either absent from or highly divergent compared with animals and microbes. These assemblies are dynamic, membrane‐embedded, and environmentally responsive, making them among the most challenging and rewarding targets in structural biology.

### Photosynthesis: Structure in action

Photosynthesis remains the quintessential plant‐specific process, and its study has already yielded some of the most iconic structures in biology. High‐resolution models of photosystem I, photosystem II, and the cytochrome *b6f* complex have defined how light energy is captured and converted into chemical energy (Naschberger et al., 2022; Sarewicz et al., 2023). More recent cryo‐EM studies of light‐harvesting complexes (LHC) and supercomplex assemblies have revealed organizational principles that regulate energy flow (Wu et al., 2023).

Yet, many central questions remain unanswered (Eckardt et al., 2024). Foremost among these is the dynamic regulation of photosynthesis under fluctuating light. Protective processes such as non‐photochemical quenching and state transitions depend on LHCII–LHCII interactions and reorganizations within the LHCII network, but the structural basis of these interactions remains unresolved. Similar challenges apply to the thylakoid membrane as a whole, where densely packed protein complexes must remain mobile and responsive to environmental change. Addressing these dynamics will require *in situ* and time‐resolved approaches, including cryo‐ET and time‐resolved cryo‐EM.

Beyond chloroplasts, photosynthetic diversity across plant lineages offers a rich structural landscape. Variations in antenna composition, pigment types, and electron transport components have evolved in response to different ecological pressures. Comparative structural studies across C3, C4, and CAM plants, or between algae and higher plants, could illuminate how structural innovations underlie adaptation to distinct environments.

### Plant signaling: Perception and response

Plant growth and development are orchestrated by an extensive network of signaling pathways, many of which rely on receptors and complexes with no direct counterparts in animals. In plants, RLKs mediate responses to developmental cues and environmental stimuli. Although some RLK ectodomains have been crystallized (Hohmann & Hothorn, 2019), their full‐length structures, including transmembrane and intracellular kinase domains, remain elusive. Similarly, hormone perception mechanisms, such as brassinosteroid recognition by BRI1 or auxin sensing by TIR1/AFB, are only partially understood in structural terms (Calderon‐Villalobos et al., 2010; Hothorn et al., 2011).

The key challenge is to visualize how ligand binding triggers conformational changes that propagate across domains and membranes to initiate signaling cascades. Such insights are particularly urgent for plant hormone pathways, which control everything from cell elongation to stress responses. Structural studies of multiprotein signaling complexes, ideally in native membranes or *in situ*, will be essential to move beyond isolated receptor domains.

Another frontier lies in the dynamic assemblies that underlie stress signaling. Calcium‐dependent protein kinases, MAP kinases, and other hubs often form transient complexes that are difficult to capture biochemically. Cryo‐EM and integrative approaches may make it possible to visualize these assemblies in the near future, providing a structural framework for understanding plant plasticity.

### Cell wall synthesis and remodeling

The plant cell wall is a defining feature of plant life, providing mechanical strength, mediating cell–cell interactions, and serving as a barrier against pathogens. At its core lies cellulose, synthesized by large rosette‐shaped cellulose synthase complexes (CSCs) embedded in the plasma membrane (Haigler & Roberts, 2019). High‐resolution structures of individual cellulose synthase isoforms have been determined, but how they assemble into functional rosettes and how these rosettes interact with the cytoskeleton and other cell wall‐modifying enzymes is not yet known.

Beyond cellulose, the synthesis and modification of hemicelluloses and pectins involve large families of glycosyltransferases and polysaccharide‐modifying enzymes, most of which are structurally uncharacterized. Capturing these enzymes in action will be crucial for understanding how the complex matrix of the cell wall is built and remodeled during plant growth, differentiation, and stress. Moreover, *in situ* studies of cell wall synthesis could reveal how multiple enzymes are spatially coordinated at the apoplast, a level of organization that goes far beyond single protein structures.

### Organelle biology and inter‐organellar communication

Plant cells are remarkable for their rich organelle diversity and their intricate networks of communication between compartments. Chloroplasts, mitochondria, and peroxisomes cooperate in photorespiration and other metabolic pathways, yet, the structural principles of these interactions are just being discovered (Midorikawa et al., 2022). Contact sites between organelles, where membranes come into close proximity to allow metabolite exchange, represent a new frontier for *in situ* structural biology.

Protein import into plastids is another major challenge. The import of thousands of nuclear‐encoded proteins through the translocon complexes at the outer and inner chloroplast envelope membranes (TOC/TIC) underpins plastid biogenesis and function, and recent studies are beginning to reveal how these assemblies are structurally organized in plants (Liang et al., 2024). High‐resolution structures of TOC/TIC components, ideally within the native membrane, would provide critical insights into how selectivity and regulation are achieved. Similar questions apply to mitochondrial protein import complexes, which in plants have diverged from their yeast and animal counterparts.

Vacuoles, too, remain largely unexplored structurally, despite their central roles in storage, signaling, and cell homeostasis. Understanding how tonoplast transporters, channels, and pumps are organized could provide new perspectives on how vacuoles contribute to plant physiology.

### Plant immunity: Resistosomes and beyond

Plant immunity offers another rich structural landscape. Intracellular immune receptors of the nucleotide‐binding leucine‐rich repeat (NLR) family detect pathogen effectors and oligomerize into large signaling complexes known as resistosomes. The cryo‐EM structure of the ZAR1 resistosome represented a landmark achievement, revealing how pathogen perception triggers assembly of a membrane‐associated pore complex (Wang et al., 2019). Yet, ZAR1 is only one member of a vast and diverse family, and whether other NLRs adopt similar or distinct architectures remains unknown.

Beyond NLRs, structural insights are needed into the complexes formed by RLKs, co‐receptors, and downstream signaling components that orchestrate defense responses. Pathogen effectors themselves, many of which target host proteins to suppress immunity, remain structurally uncharacterized. Capturing host–pathogen complexes at high resolution could illuminate the molecular arms race that underlies plant–microbe interactions.

### Evolutionary diversity and adaptation

Finally, plant structural biology has much to contribute to evolutionary biology. Plants and their algal ancestors have evolved unique solutions to fundamental cellular challenges, from energy capture to stress resilience. Comparative structural studies can reveal how conserved complexes have been adapted to plant‐specific functions. For instance, ATP synthase in chloroplasts is mostly monomeric and excluded from curved regions, in contrast to mitochondrial ATP synthase, which forms dimeric rows that drive membrane curvature (Daum et al., 2010). Similarly, variations in photosystem composition and organization across taxa illustrate how structural innovations underlie ecological adaptation.

Studying non‐model plants, including crops, extremophiles, and basal lineages, will broaden the scope of structural biology beyond Arabidopsis and illuminate the diversity of plant molecular machines. Such work will require both technical flexibility and a willingness to engage with organisms that are less genetically tractable but structurally informative.

## FROM STRUCTURES TO LANDSCAPES

The most profound challenge for plant structural biology in the coming decade is not simply to solve more structures, but to expand the very scope of what a structure represents. Traditional structural biology has delivered static snapshots, highly detailed but often divorced from the cellular and environmental context in which proteins operate. For plants, whose molecular machines are finely tuned to developmental stage, cell type, and external conditions, this static view is particularly limiting. The future lies in further moving from isolated snapshots to dynamic structural landscapes that capture heterogeneity across time, space, and environment (Figure 1).

### Capturing temporal dynamics

Time is a central dimension of plant life. Photosystems must adapt to fluctuating light within seconds; hormone signaling can reprogram transcription within minutes; immune responses must be triggered rapidly upon pathogen recognition. Static structures reveal possible conformations, but they cannot by themselves explain how transitions occur. Time‐resolved approaches, including SFX or TrCryo‐EM, hold the potential to visualize intermediate states along functional pathways. The challenge is to make these approaches routine for plant systems. This will require technical innovation, such as light‐triggered vitrification to capture photosynthetic reactions at defined timescales or microfluidic devices for rapid hormone application before plunge‐freezing. It will also require computational advances to sort and classify heterogeneous particle populations, reconstructing dynamic trajectories from mixtures of states. Success would allow us to move from molecular stills to molecular movies, illuminating how plant proteins actually function in real time.

### Embracing spatial complexity

Space is equally important. Plant cells are large, structurally complex, and highly compartmentalized. The same protein may behave differently in a mesophyll chloroplast compared with a guard cell chloroplast, or in the context of a developing versus mature tissue. *In situ* structural biology, particularly cryo‐ET, offers a path to capture this spatial diversity.

Looking ahead, the vision is of structural biology that can be performed in specific contexts: in guard cells responding to drought, in root hairs interacting with symbionts, or in developing seeds under nutrient stress. This would allow us to connect structural insights directly to physiological outcomes.

### Environmental responsiveness

Unlike many animal systems, plant proteins are highly responsive to environmental conditions. Light, temperature, water, and biotic interactions all influence protein conformation, complex assembly, and localization. Capturing structures under these variable conditions is therefore critical to understand their true biological function.

A grand challenge is to develop workflows that allow structural studies under controlled but physiologically relevant perturbations. For example, plants could be shifted between light and dark before rapid cryo‐fixation, enabling structural snapshots of photosynthetic transitions. Exposure to pathogen effectors or abiotic stresses could be coupled with structural analysis of the corresponding defense or stress–response complexes. Ultimately, linking structural changes to environmental stimuli will allow plant structural biology to move beyond abstraction and into the realm of functional ecology.

### Cross‐scale integration

Finally, structural landscapes must be integrated across scales. At one end, atomistic resolution reveals catalytic mechanisms and conformational changes. At the other, cellular and tissue ultrastructure shapes how complexes are arranged and interact. Bridging these scales is not only a technical challenge but also a conceptual one, requiring frameworks that integrate molecular detail with systems‐level behavior.

One promising direction is the integration of structural biology with systems‐level modeling. Structures of signaling complexes can inform network models of hormone responses; structural maps of photosynthetic complexes can be linked with physiological models of energy capture. Coupled with omics data, these approaches can create multiscale descriptions of plant function that are anchored in molecular reality.

## OUTLOOK

The transition from static structures to structural landscapes represents a paradigm shift for plant biology. Thus, the coming decade will be decisive for plant structural biology. The tools now at our disposal, high‐resolution cryo‐EM, tomography, XFELs, and AI‐driven prediction, make it possible to address questions that were once out of reach. We are entering a period when the molecular machines of photosynthesis, cell wall synthesis, signaling, and immunity can be studied not only in isolation but also in action, *in situ*, and in the context of whole cells and tissues. This convergence of methods offers an unprecedented opportunity to transform our understanding of plants at the molecular level.

Realizing this potential, however, requires more than technological advances. It demands strategic investments in infrastructure and training. Dedicated cryo‐EM facilities for plant biology, with staff experienced in handling plant‐specific challenges such as thick cell walls, membrane proteins, and glycosylated complexes, would lower barriers to entry for the wider community. Access to XFEL beamlines and advanced cryo‐ET workflows must be expanded beyond specialized consortia if plant researchers are to use these methods routinely. Just as importantly, young scientists must be trained in both structural and plant biology, fostering a generation of researchers fluent across disciplinary boundaries.

Collaboration will be critical. Many of the most pressing challenges in plant structural biology will not be solved by individual labs working in isolation. Coordinated networks that bring together expertise in genetics, physiology, structural biology, and computation will be needed. Shared databases of plant protein structures, interaction maps, and experimental pipelines could accelerate progress, much as similar resources have transformed biomedical research.

The implications of success extend far beyond academic knowledge. A detailed molecular understanding of photosynthesis could guide efforts to engineer more efficient light use in crops. Structural insights into hormone receptors and signaling pathways could inform strategies for improving stress tolerance or nutrient use efficiency. Deciphering the architecture of cell wall biosynthetic machinery could unlock new routes to sustainable bioenergy and biomaterials. In the context of climate change and global food insecurity, these applications underscore why investment in plant structural biology is not a luxury but a necessity.

Ultimately, the vision is clear: to move from static catalogs of plant protein folds toward dynamic, contextualized structural landscapes that explain how plants function, adapt, and survive. Meeting this challenge will require imagination, persistence, and collaboration across disciplines. But the rewards, both in fundamental knowledge and in practical applications, are immense. By revealing the molecular logic of the green world, plant structural biology can play a central role in addressing some of the most urgent challenges of the 21st century.

## CONFLICT OF INTEREST STATEMENT

The authors declare that they have no known competing financial interests or personal relationships that could have influenced the work reported in this paper.

## Data Availability

Data sharing not applicable to this article as no datasets were generated or analysed during the current study.
